# FOS protein expression and role of the vagus nerve in the rat medullary visceral zone in multiple organ dysfunction syndrome caused by subarachnoid hemorrhage

**DOI:** 10.3892/etm.2012.770

**Published:** 2012-10-26

**Authors:** YAN HE, QIANG-CHUAN QU, BANG-XING WANG, FENG-YI DU, ZHI-HONG GUO

**Affiliations:** 1 Department of Neurology, Provincial Hospital Affiliated to Shandong University, Jinan, Shandong 250021;; 2 Department of Neurology, Second Affiliated Hospital of Shandong University of Traditional Chinese Medicine;; 3 Department of Neurology, Qilu Hospital of Shandong University, Jinan, Shandong 250012 P.R. China

**Keywords:** subarachnoid hemorrhage, multiple organ dysfunction syndrome, medullary visceral zone, vagus nerve, FOS protein

## Abstract

This study was designed to observe the role of FOS protein expression in the rat medullary visceral zone (MVZ) in multiple organ dysfunction syndrome (MODS) caused by subarachnoid hemorrhage (SAH), with and without severing the vagus nerve. We also investigated the regulatory and control mechanisms of the MVZ and the vagus nerve in MODS following SAH. A model of MODS following SAH was established by injecting arterial blood into the Willis’ circle. The vagus nerve was cut off and blocked. The FOS protein expression in the MVZ was detected by immunohistochemistry. The positive expression levels of FOS in the MVZ in the SAH and SAH + severed-down vagus nerve (SDV) groups were higher than those in the normal control, sham surgery and SDV groups (P<0.01). However, expression in the SAH+SDV group was lower than that in the SAH group (P<0.01). Inflammatory damage was observed in each visceral organ at every time-phased point in the SAH group and the SAH+SDV group. The most apparent damage was at 24–36 h, consistent with the peak of FOS protein expression; the SAH+SDV group presented a greater level of damage. The inflammatory changes in surrounding visceral organs following SAH correlated with FOS protein expression in the MVZ, which indicates that the MVZ participates in the functional control of surrounding visceral organs following SAH. Severing the subphrenic vagus nerve increases the incidence of MODS following SAH and enhances SAH-induced inflammatory damage to the surrounding visceral organs, which indicates that the vagus nerve plays a role in the protection of the surrounding visceral organs in MODS following SAH.

## Introduction

Multiple organ dysfunction syndrome (MODS) is defined as simultaneous or sequential dysfunction of two or more organs or systems and normally develops following an acute and severe injury. It develops as follows: primary injury, stress response of the body, systemic inflammatory response syndrome (SIRS) and the dynamic evolution process of MODS (1), wherein SIRS is the main cause of MODS (2). Since MODS was identified in 1991, scholars in the field of trauma surgery have concentrated on studying and investigating the disorder. Studies into traumatic and infectious MODS have accomplished notable achievements (3,4); however, not enough attention is paid to cerebrogenic multiple organ dysfunction syndrome (CMODS), in the field of neurology. CMODS, an important branch of MODS, may be caused by a variety of cerebral injuries (5). CMODS caused by acute cerebrovascular diseases (ACVD) have the highest incidence of approximately 10.7% and a mortality rate of 58.4%. Compared with MODS induced by other causes, organ and system damage in CMODS occurs more rapidly, with a higher mortality rate (6,7). At present, MODS is reported as a clinical phenomenon or a complication of ACVD, with very few studies researching the disorder. In a previous study, we investigated the expression of endotoxin and inflammatory factors in a model of CMODS caused by ischemic cerebrovascular disease, as well as the function of the hypothalamus-hypophysis axis of three target glands (adrenal gland, thyroid gland and the gonads) (7). However, the neural control mechanism of CMODS has not yet been clarified.

Previous studies (8,9) have shown that when ACVD occurs, the hypothalamus plays a central role in the functional control of the neuroendocrine system. It not only participates in neuroendocrine-immune modulation through the hypothalamus-hypophysis-target gland axis and the hypothalamus-spinal cord-sympathetic nerve axis, but may also act through the hypothalamus-medullary visceral zone (MVZ)-vagus nerve route. As an important hub for regulating visceral activity and as a relay station for neuro-immunomodulation (10), the MVZ (11), an arc-shaped band from the dorsomedial to ventrolateral area in the middle-caudal segment of the medulla oblongata, is involved in the cholinergic anti-inflammatory pathways of the vagus nerve and plays an important role in MODS (12).

At present, MODS therapy includes protopathy, anti-infective treatment, immune regulation, organ support and protection, but with a limited effect and a high mortality rate (13). As a result, the search for effective treatment methods is continuing. The discovery of the anti-inflammatory effects of the cholinergic anti-inflammatory pathways undoubtedly provided new ideas and methods for the treatment of MODS (14–16). This study aimed to observe the expression of FOS protein in the rat MVZ following subarachnoid hemorrhage (SAH) and the severing of the celiac branches of the vagus nerve, as well as provide experimental evidence to clarify the neuroendocrine and immunological mechanisms of MODS caused by ACVD.

## Materials and methods

### Animal grouping

Adult male Wistar rats (n=90, clean grade), each weighing 250–300 g, were provided by the Experimental Animal Center of Shandong University, China. Rats were divided into 15 groups as follows: the normal control group; the sham surgery group; the severed-down vagus nerve group (SDV group); the subarachnoid hemorrhage group (SAH group), which was equally divided into 6 subgroups at the time-phased points of 4, 12, 24, 36, 48 and 72 h; and the subarachnoid hemorrhage and severed-down vagus nerve group (SAH+SDV group), which was also divided into 6 subgroups in the same way. Each of the 15 groups contained 6 rats. The animal use protocols used were approved by the Institutional Animal Care and Use Committee at Shandong University (Shandong, China). All animal experiments were carried out in accordance with the National Research Council Guide for the Care and Use of Laboratory Animals, as adopted by the National Institutes of Health.

### Preparation of the animal model

The SDV procedure was as follows: the rats were given a laparotomy after being narcotized with 1% pentobarbital solution. The right and left vagus nerves were exposed in the lesser curvature of the stomach, ligated with silk thread and cut off ∼5 mm away from every branch of the vagus nerve (gastric, hepatic and celiac branches). The postoperative animals were given solid food and those in the SAH+SDV group were given the SAH surgery after four weeks (17).

The sham surgery was performed at the same time as the SDV and SAH surgeries. Right and left vagus nerves were exposed in the SDV surgery, without ligation or cutting. In the SAH surgery, 100 μl normal saline was injected by intubation.

### Inspection items

Postoperative vital signs were observed at every time-phased point, at which femoral vein blood was assayed by routine blood tests, liver function tests [alanine transaminase (ALT) and aspartate transaminase (AST)], renal function tests [blood urea nitrogen (BUN), serum creatinine (Cr)] and measurement of myocardial enzyme levels [creatinine kinase (CK)]. The brain, lung, liver, kidney and small intestine were monitored for pathological changes. FOS protein expression in frozen brain sections from the MVZ was assayed using the immunohistochemical ABC method (18).

### Diagnostic criteria of SIRS and MODS

Diagnosis of SIRS and MODS was performed according to the diagnosis criteria of SIRS and MODS in experimental animals proposed by Bone *et al*(1).

### Statistical analysis

All indexes were expressed as the mean ± standard deviation (SD) and processed by SPSS 10.0 software with Student’s t-test and variance analysis. P<0.05 was considered to indicate a statistically significant difference.

## Results

### Animal model

**Figure 1:**
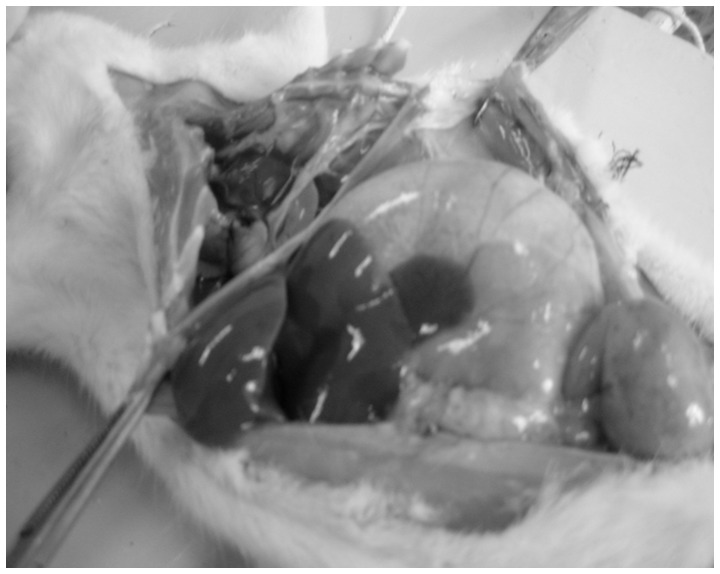
Severe flatulence appeared in rats after cutting the vagus nerve in the SDV group. SDV, severed-down vagus nerve.

In the sham surgery and SDV groups, animals expressed mild listlessness and increased respiration within 12 h of surgery, which improved after 12 h. Abdominal distension, slightly reduced feeding and normal activities were observed in the SDV group. In the SAH group there were 11 cases (30.6%) of postoperative coma, cyanosis and hyperventilation; 19 cases (52.8%) of epileptic seizure, with listlessness, loss of appetite, increased respiration and a slowed reaction time to pinprick pain in postoperative woken rats and 14 cases (38.9%) of mortality prior to the time-phased points. In the SAH+SDV group, all rats displayed serious abdominal distension (Fig. 1). There were also 14 cases (38.9%) of postoperative coma, cyanosis and hyperventilation; 20 cases (55.6%) of epileptic seizure, with listlessness, loss of appetite, increased respiration and a slowed reaction time to pinprick pain in postoperative woken rats and 17 cases (47.2%) of mortality prior to the time-phased points.

Compared with the normal control group, there was no significant difference in breathing rate, heart rate, body temperature or white blood cell count (WBC), ALT, AST, BUN, Cr and CK of peripheral blood between the sham surgery group and the SDV group (P>0.05). The above parameters were higher in the SAH group and the SAH+SDV group than those in the normal control, sham surgery and SDV groups (P<0.01). There was no significant difference in breathing rate, heart rate or CK between the SAH+SDV group and the SAH group (P>0.05); however, there was a significant difference in body temperature and WBC, ALT, AST, BUN and Cr of peripheral blood between the SAH+SDV group and the SAH group, with the SAH+SDV group producing significantly higher results than the SAH group (P<0.01).

### Pathological changes

Organ and tissue structure in the normal control group and sham surgery group was essentially normal; however, in the sham surgery group the alveolar septa was broadened, capillaries were congested with blood and some neutrophil granulocytes infiltrated the lung tissue. The lung and kidney tissues in the SDV group were the same as in the sham surgery group. We noted edema and thickening in the mucous layer in the small intestine, and a cloudy swelling of hepatocytes appeared in the hepatic tissue in the SDV group.

The pathological changes of the surrounding visceral organs at every time-phased point in the SAH group included considerable nonspecific inflammation. In lungs the following changes were observed: angiotelectasis and congestion, endovascular neutrophilia as well as intra-alveoli and perivascular exudation appeared at 4 h; the alveolar cavity was expanded and fibrin was exuded in bronchial lumen and tracheal lumen at 12 h; and mild and chronic lymphocytic bronchopneumonia occurred at 24 and 36 h. These inflammatory responses were relieved at 48 h. Inflammatory cell infiltration and alveolar expansion appeared at 72 h. In the small intestine, the following changes were observed: edema and thickening in the mucosa and submucosa appeared at 24 h; edema, thickening and congestion between the muscular layer and mucous layer as well as light inflammatory cell infiltration occurred at 36 and 48 h; and congestion and edema appeared in the mucosa and submucosa at 72 h. In the liver, the following changes were observed: a number of liver cells were swollen at 8 h; spotty necrosis of liver cells, liver sinus expansion and markedly swollen liver cells around the central vein and individual ballooning degeneration occurred at 24 h; chronic inflammatory cell infiltration and acidophilic degeneration of liver cells appeared in stroma at 36 h; and acidophilic degeneration of liver cells disappeared at 48 h. In the kidney, the following changes were observed: the renal interstitium was hyperemic, a number of proximal tubular epithelial cells were swollen and the cytoplasm became empty at 12 h; an atrophied glomerulus, renal tubular expansion and swollen proximal tubular epithelial cells appeared at 24 and 36 h; individual necrosis of the renal proximal tubule appeared at 48 h; and no changes occurred at later time-phased points.

The inflammatory pathological changes of the small intestine, liver and kidneys at each time-phased point in the SAH+SDV group were more apparent and lasted longer than those in the SAH group. The changes that appeared in the lungs at 4 h (angiotelectasis, congestion and endovascular neutrophilia) were more evident at 24 and 36 h. A number of animals showed signs of lobar pneumonia at 48 h and inflammatory cell infiltration and alveolar expansion were still present at 72 h. The inflammatory changes observed in the small intestine (edema and thickening in mucosa and submucosa) at 4 h and at each time-phased point were more apparent in the SAH+SDV group than in the SAH group. The congestion and edema in the mucosa and submucosa and lymphocyte infiltration remained at 72 h. Inflammatory lesions of the liver lasted longer in the SAH+SDV group than in the SAH group and lymphocyte infiltration remained at 72 h. Kidney damage was more apparent in the SAH+SDV group than in the SAH group. Protein and cellular casts appeared in the proximal tubule at 36 h and proximal tubular epithelial cells remained swollen at 72 h.

### Incidence of SIRS and MODS in the SAH and SAH+SDV groups

The incidence of SIRS following SAH in rats was 100%. There were 25 cases with MODS in the SAH group, with an incidence of 69.4%. Fourteen animals were dead prior to the time-phased point, accounting for 38.9% in the SAH group. There were 27 cases with MODS in the SAH+SDV group, with an incidence of 75.0%. Seventeen animals were dead prior to the time-phased point, accounting for 47.2% in the SAH+SDV group.

### Positive protein expression of FOS

**Figure 2:**
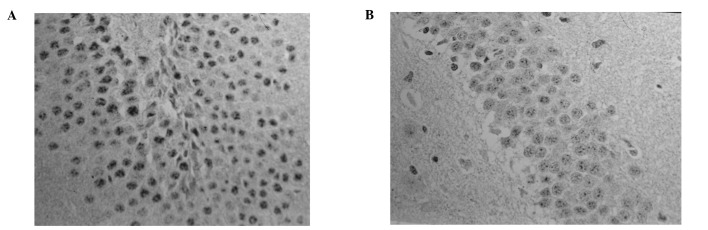
FOS expression levels were lower in the MVZ of the SAH+SDV group than in the SAH group. FOS expression in the MVZ at 24 h in (A) the SAH group and (B) the SAH+SDV group using immunohistochemistry (x200). MVZ, medullary visceral zone; SAH, subarachnoid hemorrhage; SDV, severed-down vagus nerve.

A number of hypochromatic FOS-positive cells were found in the MVZ in the normal control and sham surgery groups; however, more FOS-positive cells were found in the MVZ in the SDV group, but the difference was not statistically significant (P>0.05). The number of FOS-positive cells in the MVZ in the the SAH and SAH+SDV groups was significantly higher than those in the normal control, sham surgery and SDV groups (P<0.01); however, the number of FOS-positive cells in the MVZ in the SAH+SDV group was significantly lower than that in the SAH group (P<0.01; Table I). In the SAH group, FOS-positive cells were almost hyperchromatic, with a concentrated distribution in the nucleus tractus solitarius (NTS) of the dorsomedial area (DM), the dorsal motor nucleus of the vagus nerve (DMV) and the lateral reticular nucleus (LRN) between the area postrema (AP) and the ventrolateral medulla (VLM). In the SAH+SDV group, FOS-positive cells were mainly distributed in the NTS and LRN. The expression level peaked at 24 h and then gradually reduced at 36–48 h, but still existed at 72 h (Fig. 2).

## Discussion

ACVD complicated with MODS is common in the clinic; however, there are very few studies using related animal models. This study was completed according to the diagnosis criteria of SIRS and MODS in experimental animals proposed by Bone *et al*(1). Our results correlated with the criteria for an MODS animal model, with SIRS incidence of 100%, MODS incidence of 69.4% and mortality of 38.9% following SAH. Therefore, our model of MODS following SAH, produced by injecting arterial blood into the Willis’ circle, was successful.

FOS protein in the MVZ in ACVD is the expression product of immediate early gene c-fos. As a regulation factor in cell transcription, it participates in the transcriptional processes of various effect enzymes. A number of studies (19,20) confirm that FOS protein plays a role of ‘tracer’ and may be used to locate neuronal activity and monitor function. Another study (21) found that different dosages (0.4/0.8 units) of collagenase in a rat cerebral hemorrhage model, increased the positive expression of FOS in the MVZ, compared with the levels in a control group. The number of FOS-positive cells in the 0.8 unit group was significantly higher than that in the 0.4 unit group. Therefore, serious ACVD may cause changes in FOS protein expression in the MVZ and the amount of induced FOS is consistent with the intensity of the stimulus, to a certain extent. As one of the important control centers for stress reaction, the MVZ may be related to the fibers in the hypothalamus, limbic system and surrounding visceral organs and therefore participate in the regulation of various somatic and visceral sensations and activities (including breathing, heartbeat, blood pressure, gastrointestinal motility, endocrine responses and immunoreaction) through its NTS, DMV, nucleus ambiguus (NA), ventrolateral reticular nucleus (VLR) and other internal nuclear groups. A previous study has shown (20) that electronic damage to rat NTS in the MVZ may cause neuronal pulmonary edema. It is considered that the uplink fiber of the caudal NTS has a wide fiber connection with catecholaminergic neurons in the brainstem reticular formation and that neuronal pulmonary edema may be caused by regulation of the release of catecholamine. The current study revealed that the respiratory frequency increased at each time-phased point in the SAH group compared with the control group, particularly at 24, 36 and 48 h. Nonspecific inflammatory damage in the lungs appeared 4 h after SAH, peaked at 24–36 h and gradually reduced at 48 h. At the same time, the positive FOS protein expression in the MVZ followed the same pattern. The dense positive protein expression of FOS in the caudal NTS and VLM appeared at 4 h in the SAH group, peaked at 24 h, with continuously high levels at 36 h and then gradually reduced at 48 h with some positive expression still shown at 72 h. This suggests that a series of physiological pathological changes in the brain following SAH may influence the function of the NTS in the MVZ and VLM, cause imbalance in the respiratory function of the lungs in normal conditions and lead to neurogenic pulmonary edema.

Previous studies (21,22) have shown that in a model of cardiovascular stress reaction, hypertension and acute myocardial ischemia induced by intravenous injection of pituitrin, FOS protein expression in the medulla oblongata was limited to the MVZ, with all surrounding nucleus groups displaying negative expression. FOS proteins in the MVZ were densely expressed in the NTS and VLM, 50% of which were FOS/TH (thyrosine hydroxylase) double-labeled positive neurons, which indicates that catecholaminergic neurons in the MVZ participate in the stress reaction of cardiovascular noxious stimulation. Changes in rat blood pressure and electrocardiogram following SAH were not measured in this study; however, the changes in heart rate and myocardial enzyme may reflect the rat’s myocardial ischemic condition. The dense expression of FOS protein in the MVZ of the SAH group was mainly observed in the NTS, VLM and DMV, with a reduced amount shown in the AP, at 4 h after hemorrhage and peaking at 24 h. Dense expression was still observed at 36 h, which gradually reduced at 72 h. The positive expression of FOS protein was consistent with changes in heart rate and myocardial enzyme, which indicates that the positive expression of NTS and VLM in the MVZ is related to peripheral myocardial ischemia and that the MVZ participates in the regulation of cardiac function following SAH.

Ge *et al*(23) reported a large amount of FOS protein expression in the muscle plexus and submucosal neurons of the NTS and colon wall in the MVZ of the rat model of gastrointestinal stress reaction, which indicates that NTS may participate in the gastrointestinal stress reaction by influencing the activity of the colon muscle plexus and submucosal neurons. In the current study, rats in the SAH group suffered inflammatory damage to the intestine 24 h after SAH, which peaked at 36–48 h and was significantly relieved at 72 h. The consistent expression of FOS in the MVZ following SAH, particularly the dense expression of FOS in the DMV, indicates that inflammatory damage to the intestinal mucosa and submucosa is a result of influence on the function of the NTS and DMV following SAH.

Studies (24,25) have shown that, similar to MODS caused by trauma, infection and shock, SIRS is also an important pathological basis of ACVD complicated with MODS and adjustment of the nerve-endocrine-immune system is a fundamental cause of ACVD with MODS. Additionally, a number of studies found that as well as the hypothalamus-pituitary-target gland axis and the hypothalamus-spinal cord-sympathetic nerve axis, the hypothalamus-MVZ-vagus nerve is another nerve-endocrine-immune route (25). Borovikova *et al*(26) found that lipopolysaccharides (LPS) and cytokines stimulate the anti-inflammatory pathway of the hypothalamus-pituitary-adrenal gland axis after activating the afferent vagus nerve fiber. As a main transmitter in the vagus nerve, acetylcholine (ACh) significantly reduces inflammatory factors [tumor necrosis factor, (TNF) and interleukin (IL)-1D, IL-6, IL-18] secreted by human macrophages. Furthermore, severing the subphrenic vagus nerve in rats challenged with LPS significantly weakens the adrenocorticotrophic hormone reaction and prevents hypothalamic norepinephrine activation and fever reaction aggravating animal injuries (27). Yang *et al*(10) injected LPS into rat abdominal cavities and found a large amount of FOS expression in the cerebral frontoparietal cortex, limbic forebrain, hypothalamic paraventricular nucleus (PVN), supraoptic nucleus (SON) and the MVZ. The current study revealed that FOS expression in the MVZ induced by LPS decreased after severing the subphrenic vagus nerve, which indicates that peripheral immunologic information may be transmitted though the vagus nerve-MVZ to the hypothalamus and other control regions. Additionally, the positive expression of FOS in the MVZ in the SAH+SDV group was significantly reduced compared with the SAH group, which indicates that severing the subphrenic vagus nerve may prevent the inflammatory information of abdominal visceral organs from being transmitted to the control regions when MODS occurs following SAH.

To date, studies have focused on how humoral factors or autocrine and paracrine factors influence the release of anti-inflammatory mediators or pro-inflammatory mediators when SIRS occurs (28); however, the anti-inflammatory effects of nervous-endocrine anti-inflammatory pathways (i.e., the vagus nerve and its transmitter ACh) have been rarely studied. In fact, compared with the humoral anti-inflammatory pathways, cholinergic anti-inflammatory pathways, with shorter reaction times, quickly and directly regulate the systemic inflammatory responses and inhibit the lethal effect of biotoxin (29). Tracey *et al*(27) observed *in vitro* that ACh has a stronger inhibitory effect on TNF-α as well as the release of inflammatory factors IL-1, IL-6 and IL-18 generated as a result of macrophage stimulation by LPS. Hansen *et al*(30) found that severing the subphrenic vagus nerve in endotoxic shock rat models results in increased shifts of intestinal flora and tolerance of endotoxin. Furthermore, the levels of endotoxin, IL-1 and IL-6 in the blood significantly increased following intraperitoneal injection of LPS. In the SAH group and the SAH+SDV group, the incidence of SIRS was 100%; however, in the SAH group the incidence of MODS was 69.4% and animals dead prior to the time-phased points accounted for 38.9%; while in the SAH+SDV group the incidence of MODS was 75.0% and animals dead prior to the time-phased points accounted for 47.2%, significantly higher than those in the SAH group. The body temperature, WBC number, kidney function and liver function in the SAH+SDV group were significantly higher than those in the SAH group. In the SAH+SDV group, the inflammatory damage to the peripheral visceral organs including the lungs, small intestine, liver and kidneys appeared earlier, lasted longer and was more serious compared with those in SAH group. In addition, although the mean breathing rate, heart rate and myocardial enzymes in the SAH+SDV group were slightly higher than those in the SAH group, there was no significant difference beween the two groups. This may be related to the completeness of the vagus nerve pathway controlled by the visceral organs of the thoracic cavity. Therefore, severing the subphrenic vagus nerve increased the incidence of MODS following SAH and enhanced inflammatory damage to the surrounding visceral organs caused by SAH. The mechanism of this may be related to the severing of the cholinergic anti-inflammatory pathways. Our results indicate that the vagus nerve may protect surrounding visceral organs in MODS following SAH.

The mechanism of MODS caused by ACVD is complicated. In this study we found that serious inflammatory damage and SIRS appeared in rat organs, such as the lungs, small intestine and liver, following SAH and that the pathological process was consistent with the positive expression of FOS in the MVZ. Severing the subphrenic vagus nerve may increase the incidence of MODS following SAH and enhance the inflammatory damage to surrounding visceral organs caused by SAH, which indicates that SIRS is the pathologic basis of MODS following SAH. Our study showed that MVZ is involved in the functional regulation of surrounding visceral organs following SAH and is one of the direct control centers of MODS following SAH. Finally, the vagus nerve has a potential protective effect on the surrounding visceral organs in MODS following SAH.

## Figures and Tables

**Table I:** FOS protein expression in the medullary visceral zone at different time points in each group.

Group	n	Number of FOS protein-positive cells (mean ± SD, x10^−3^ μm^2^)
Normal	6	5.83±1.17
Sham surgery	6	6.33±1.21
SDV	6	7.17±1.47
SAH		
4 h	6	49.66±4.63^a–c^
12 h	6	76.50±4.72^a–c^
24 h	6	115.16±13.79^a–c^
36 h	6	90.84±12.86^a–c^
48 h	6	62.10±8.19^a–c^
72 h	6	39.67±5.24^a–c^
SAH + SDV		
4 h	6	5.51±5.01^a–d^
12 h	6	52.01±4.82^a–d^
24 h	6	93.57±10.46^a–d^
36 h	6	82.33±9.15^a–c,e^
48 h	6	64.83±7.63^a–c^
72 h	6	42.15±5.34^a–c^

a P<0.01 vs. the normal group;

b P<0.01 vs. sham surgery group;

c P<0.01 vs. SDV group;

d P<0.01, SAH+SDV vs. SAH group;

e P<0.05, SAH+SDV vs. SAH group. SD, standard deviation; SAH, subarachnoid hemorrhage; SDV, severed-down vagus nerve.
